# Chronic Osteomyelitis or Squamous Cell Carcinoma of the Mandible: A Case Report With Diagnostic Challenges

**DOI:** 10.7759/cureus.114928

**Published:** 2026-08-21

**Authors:** Abdullah M Alsoghier, Saleh Alrumayyan, Asma Almunaye, Ibrahim Alsanie, Wajdi Mohammed (Bin), Saad Hajeri

**Affiliations:** 1 Department of Oral Medicine and Diagnostic Sciences, College of Dentistry, King Saud University, Riyadh, SAU; 2 Oral Medicine Clinic, Dental University Hospital, King Saud University Medical City, Riyadh, SAU; 3 College of Dentistry, King Saud University, Riyadh, SAU; 4 Department of Maxillofacial Surgery and Rehabilitation, King Fahad Medical City, Riyadh, SAU

**Keywords:** carcinoma, case report, mouth neoplasms, osteomyelitis, squamous cell

## Abstract

It is not uncommon for many of the patients with oral squamous cell carcinoma (OSCC) to encounter diagnostic delays due to the similarity of their initial presentations with other jaw lesions. The present case report discusses jaw pain after a dental extraction, initially attributed to chronic osteomyelitis. After long and multiple diagnostic and prognostic procedures, the patient had mandibulectomy, neck dissection, and reconstruction for histopathologically substantiating stage pT4a OSCC. Delays in treatment may necessitate more aggressive interventions. Improved clinical awareness, telemedicine, enhanced doctor-patient communication, and refined diagnostic protocols contribute to better outcomes. This case highlights the diagnostic challenges of OSCC, particularly its ability to mimic chronic osteomyelitis. A delayed diagnosis emphasises the need for early recognition and intervention. While imaging aids in initial assessment, histopathology is crucial for a definitive diagnosis.

## Introduction

Oral squamous cell carcinoma (OSCC) is the most common malignant epithelial neoplasm affecting the oral cavity, accounting for more than 90% of all oral malignancies. It has a notably higher prevalence among men, tobacco users, and alcohol consumers [1,2]. OSCC most commonly manifests as an ulcer with fissured and raised exophytic margins, a lump, erythroplakia, leucoplakia, or a mixed lesion. It can also be a non-healing extraction socket or an enlarged, fixed, and hard cervical lymph node [2].

OSCC exhibits a wide range of clinical signs and symptoms, including pain, swelling, ulceration, mobile teeth, and bleeding, which are also common in oral inflammatory conditions, such as osteomyelitis [3,4]. Osteomyelitis is an inflammatory condition characterised by bone destruction, typically caused by infectious microorganisms. It can be confined to a single part of the bone or affect multiple areas, including the marrow, cortex, periosteum, and surrounding soft tissue [5]. Previous studies showed different mechanisms by which both can induce cortical perforation. For instance, perforation in osteomyelitis occurs via a cloaca in reactive periosteal bone, forming a sinus tract, with accompanying periosteal reaction rather than destructive loss [6]. In comparison, OSCC perforation results from direct tumour invasion through the Haversian system, producing an irregular destructive defect without periosteal reaction [7]. Notably, the bone resorption in both conditions is considered osteoclast-mediated, although it is infective in osteomyelitis and tumour-driven via RANKL/TNF-α in OSCC [7].

The present study reports the diagnosis and surgical management of a 62-year-old male patient with a five-month history of right jaw pain following the extraction of a lower third molar, who was initially diagnosed with chronic osteomyelitis and later with invasive OSCC.

## Case presentation

**Figure 1:**
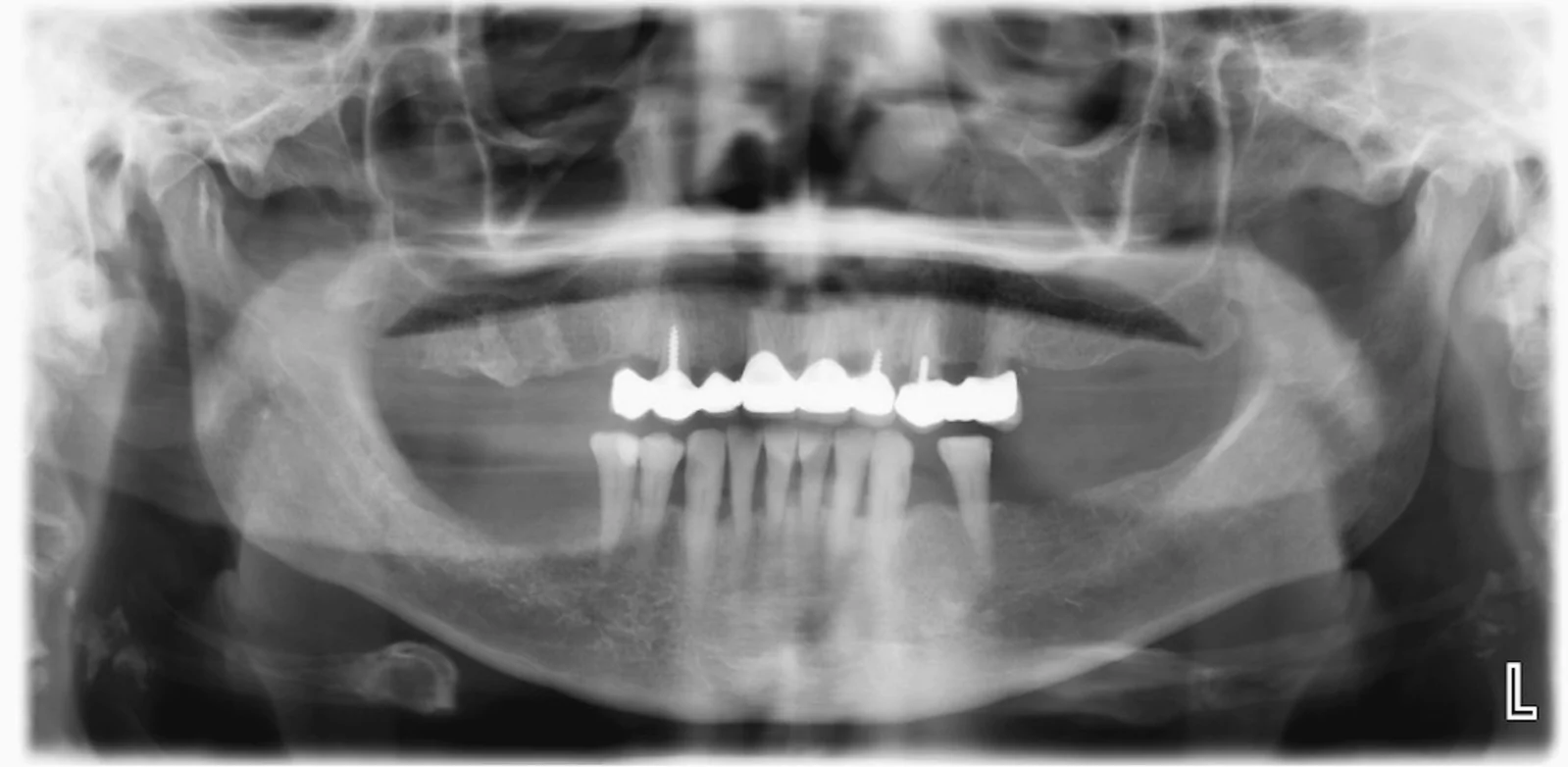
A panoramic radiograph taken on May 2024 depicting a subtle radiolucency of the alveolar crest in the right mandibular molar region

A 62-year-old Saudi man presented to the Oral Medicine specialist clinic at King Saud University Dental Hospital in May 2024, complaining of a history of jaw pain on the right side for more than five months. The pain started after extracting a badly decayed lower right third molar at a private hospital. It was described as dull, moderate in severity (5 out of 10-point pain scale), exacerbated by eating, localised, and radiating to the lower and anterior aspects of the lower right jaw and superiorly to the temporal area associated with occasional bleeding and headache on the right side extending from the angle of the mandible. The patient received a seven-day course of antibiotics, which, however, did not alleviate his symptoms. A panoramic radiograph obtained in May 2024 showed a lower definition of the alveolar crest in the right mandibular molar region, with slight radiolucency of the alveolar bone and slightly reduced bone height as compared to the contralateral side of the mandible. Due to the inherent limitations of this two-dimensional radiograph, a detailed assessment of this area was not possible. Therefore, acquisition of a cone-beam computed tomography (CBCT) scan was planned (Figure 1).

The patient reported no tooth mobility, dysphagia, dysarthria, notable weight loss, night sweats, or other frank malignancy symptoms. Medical review revealed a history of type 2 diabetes mellitus and metformin administration. No allergic reactions were observed. The patient had been using tobacco for more than 20 years and had retired.

Multi-detector computed tomography (MDCT) and magnetic resonance imaging (MRI) conducted at a private hospital in April 2024 indicated that the patient's signs and symptoms were initially attributed to chronic osteomyelitis. MDCT revealed evidence of right-sided mandibular body bone erosion and resorption in the lateral bone cortex, accompanied by adjacent soft tissue swelling and enhancement. MRI revealed an aggressive process centred on the right mandibular body, with abnormal bone marrow exhibiting low signal on T1, associated with a lateral cortical breach and periostitis, as well as evidence of enhanced soft tissue overlying the cortical breach and on the lingual surface. Despite these findings, the diagnosis of chronic osteomyelitis was deemed incorrect upon further evaluation.

**Figure 2:**
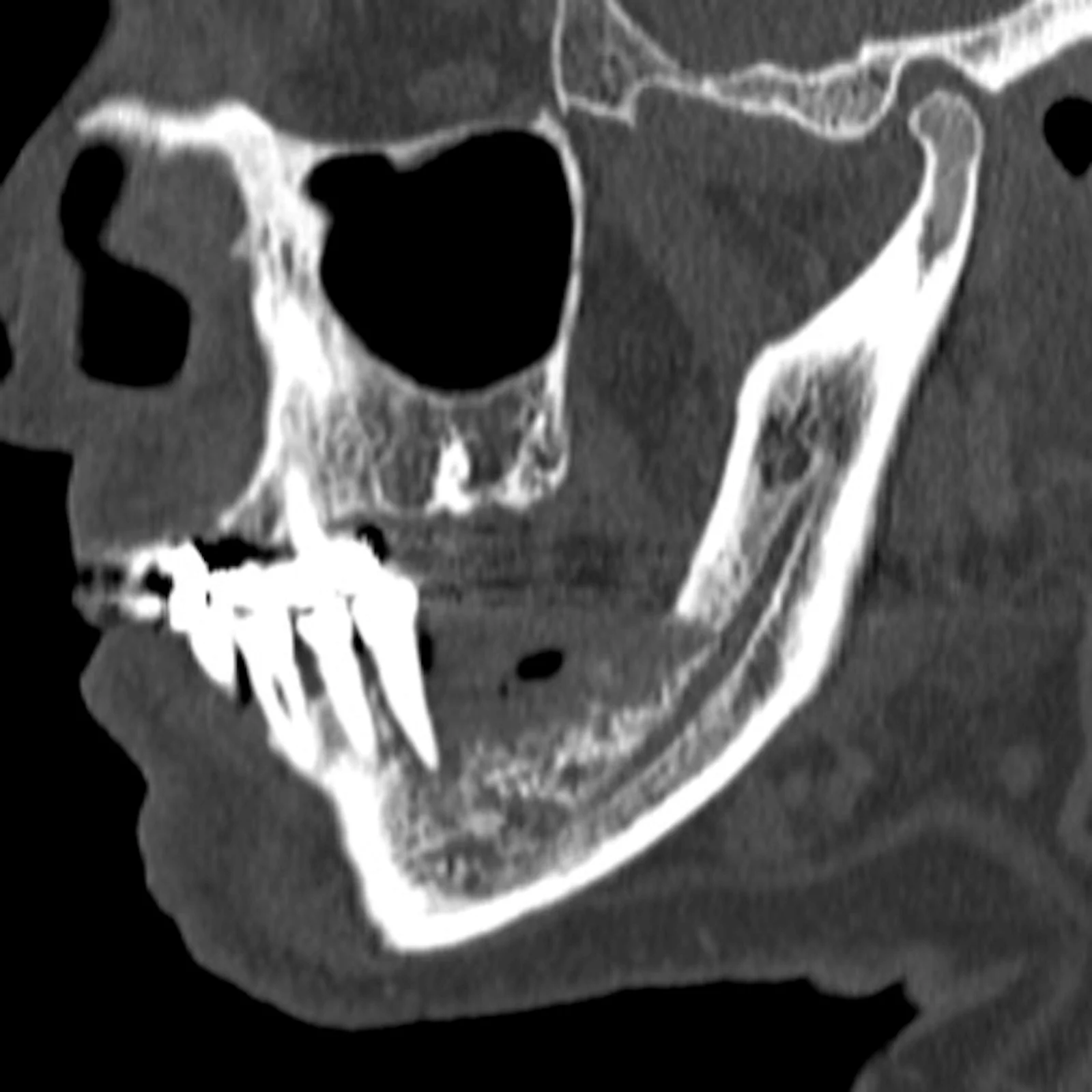
Longitudinal cross-section of the right mandible depicting the lesion

An MDCT scan dated August 2024 revealed osseous erosive changes within the right mandibular posterior molar and premolar areas. The lesion exhibited ill-defined and low-attenuation features extending anteriorly from the ascending ramus to the area of the right mandibular second premolar (as this tooth was present) anterioposteriorly. The bones at the distal and apical aspects of the tooth appeared to be eroded. The lesion also disrupted the alveolus in the superior area and extended inferiorly, abutting the superior cortical outline of the inferior alveolar canal. The involvement of the neighbouring tooth can be noted as well as the inferior extension of the lesion and its relationship with the inferior alveolar canal (Figure 2).

**Figure 3:**
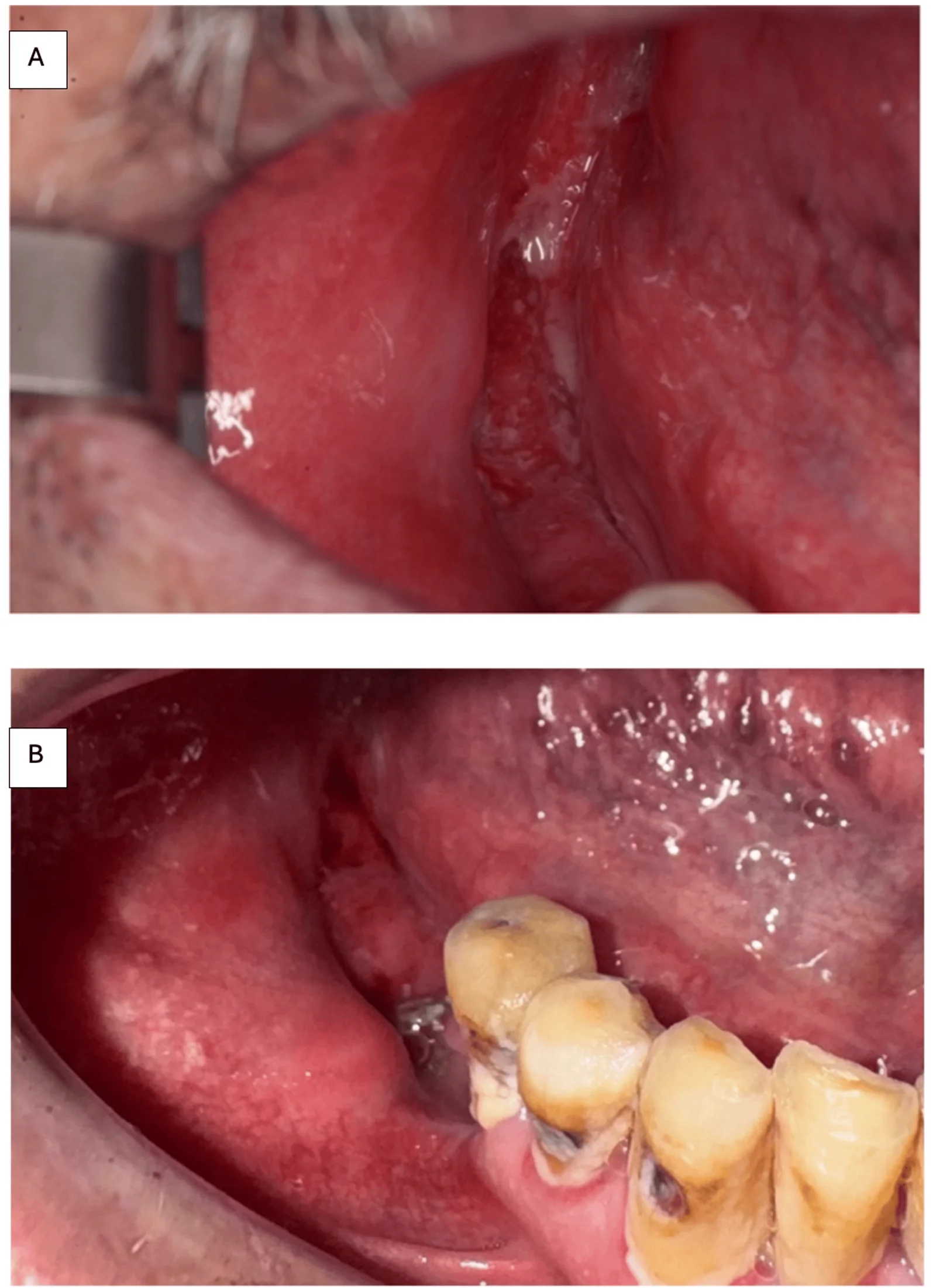
Clinical images showing (A) a non-homogeneous and erythematous patch overlying the lower right alveolar gingiva to the retromolar trigone and (B) frank bone exposure on the right alveolar ridge at the side of the extracted lower right third molar associated with poor dental hygiene

Physical examination revealed submandibular and cervical lymphadenopathies, with both sides being movable and nontender. Irregular, frank bone exposure can be observed on the right-side alveolar ridge on the side of the extracted lower right third molar, with overlying oedematous and bleeding tissue upon examination of the gingival tissue. The bone sequestra extended posteriorly to the area that was once the retromolar area and anteriorly to the site of the extracted lower right first and second molars, with overlying oedematous and erythematous gingival tissues and poor oral hygiene (Figure 3).

Due to diagnostic uncertainty between inflammatory and neoplastic processes at the time of initial assessment, a single incisional biopsy was performed of the right posterior mandibular lesion.

Histopathological assessment

**Figure 4:**
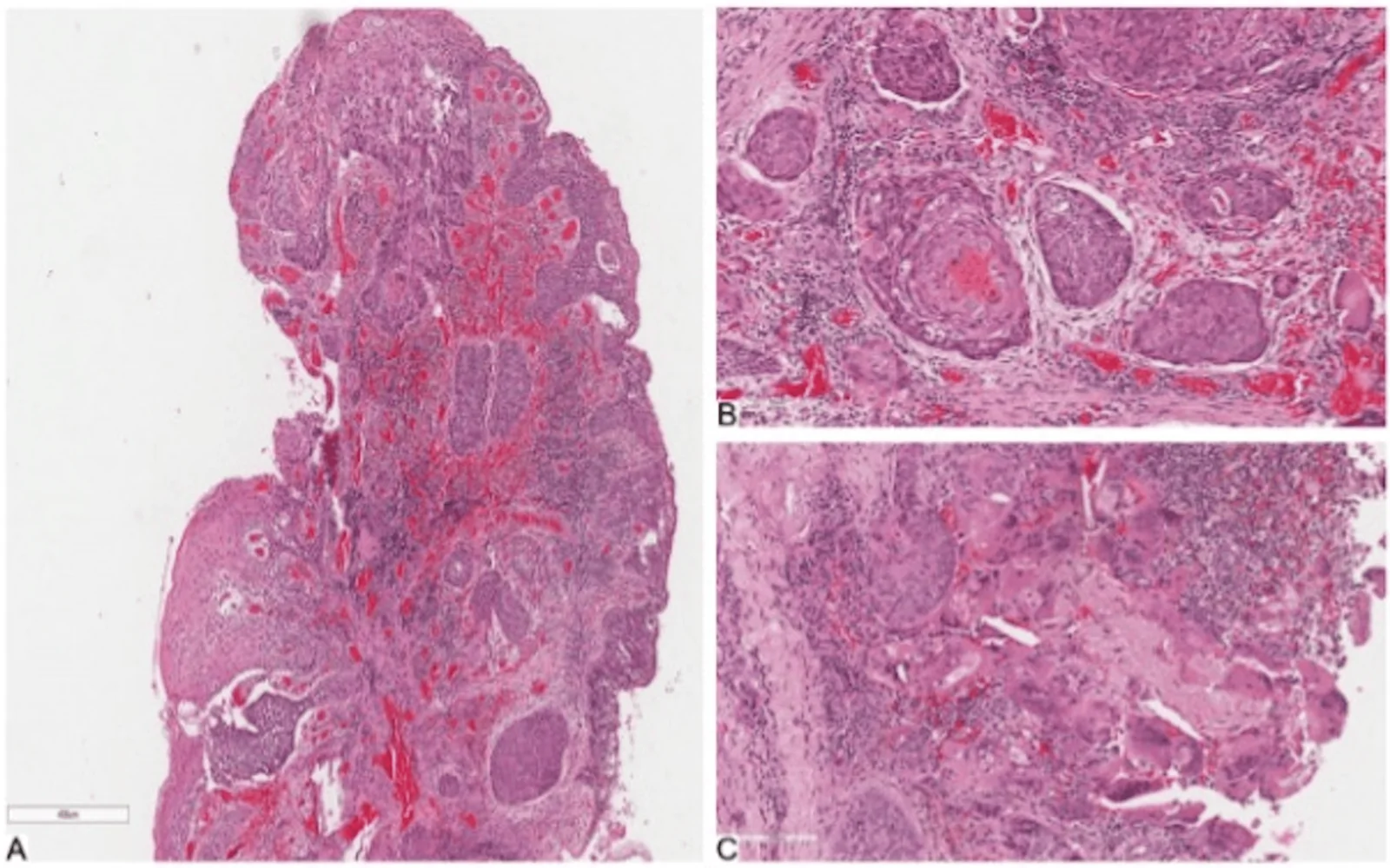
Haematoxylin and eosin-stained sections revealing the histopathology of mandibular squamous cell carcinoma. Low-power view showing (A) epithelial proliferation invading the underlying connective tissue (×5 magnification), (B) malignant squamous nests with central keratin pearl formation and pleomorphic tumour cells (×20 magnification), and (C) stromal keratin flakes eliciting a foreign body-type giant cell reaction (×20 magnification)

Microscopic examination of the incisional biopsy specimen from the right posterior mandible revealed multiple fragments of the oral mucosa with features consistent with malignant neoplasm. The surface epithelium was severely dysplastic and minimally keratinised with clear evidence of invasive growth into the underlying connective tissue and bone. The neoplastic component was composed of infiltrative islands and squamous epithelium (Figure 4A).

The malignant islands displayed a haphazard architectural pattern, stromal retraction, and central keratinisation and exhibited prominent squamous differentiation characterised by keratin pearl formation (Figure 4B). The constituent cells were large, polygonal, and eosinophilic, with marked nuclear pleomorphism and hyperchromasia. Scattered keratin flakes and pearls were noted within the tumour stroma, eliciting a foreign-body giant cell reaction (Figure 4C). The stroma itself was haemorrhagic and highly vascular, showing dilated and congested blood vessels interspersed with dense inflammatory infiltrates predominantly composed of lymphocytes, plasma cells, and multinucleated giant cells. The foci of hemosiderin-laden macrophages (siderophages) were also identified.

**Figure 5:**
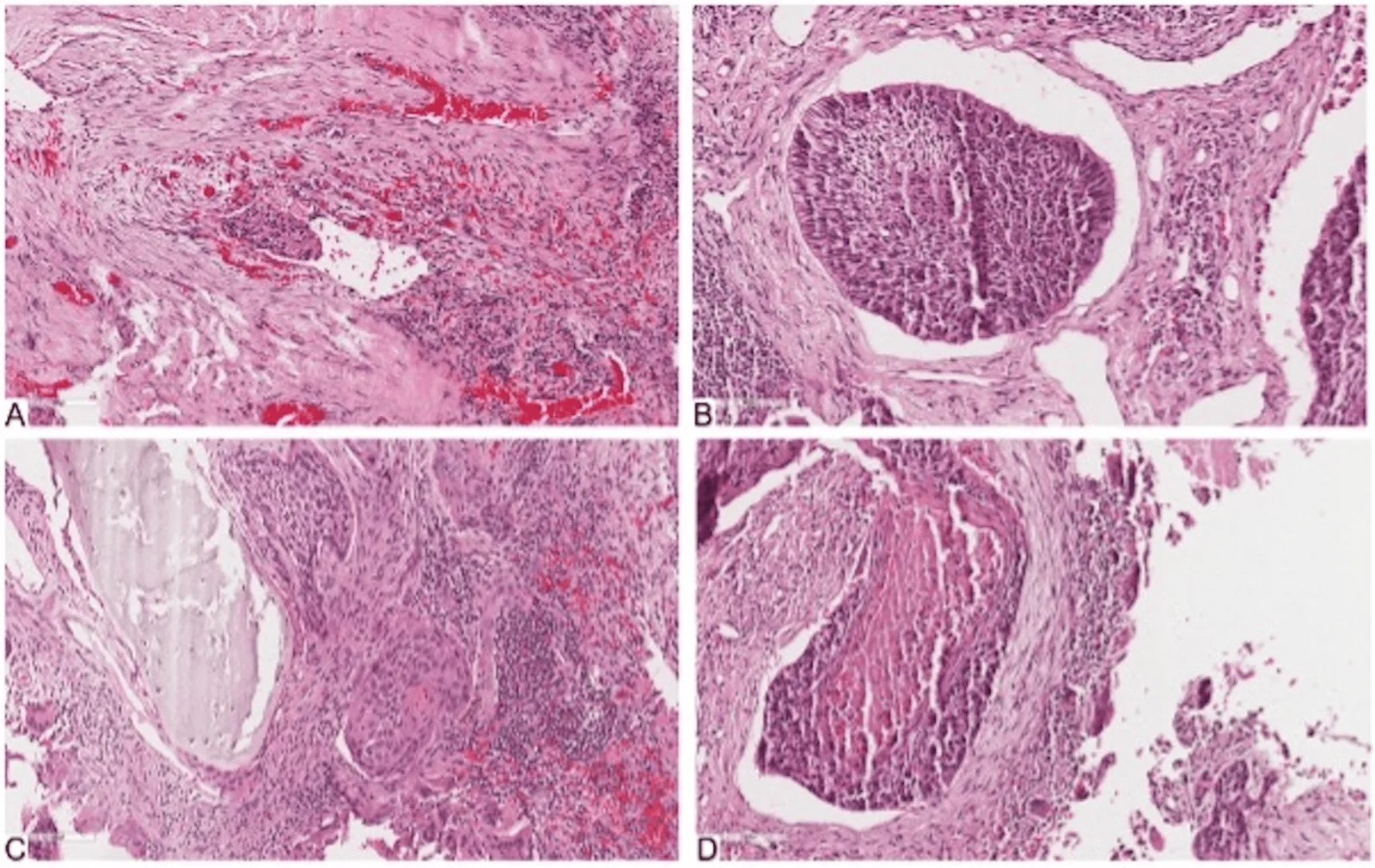
Haematoxylin and eosin-stained sections revealing the tumour invasion into lymphovascular channels and bone, along with focal comedonecrosis. Low-power view showing (A-B) malignant epithelial islands demonstrating lymphovascular invasion (×20 magnification), (C) invasion of bone with adjacent inflammation (×20 magnification), and (D) comedonecrosis and apoptotic debris in tumour nests (×20 magnification)

Lymphovascular invasion (Figure 5A-5B) and invasion of the underlying bony structures by the malignant epithelium were observed, with trabeculae showing variable viability (Figure 5C) and several areas exhibiting empty osteocytic lacunae consistent with devitalised bone (sequestrum). Comedonecrotic foci and frequent apoptotic bodies were noted (Figure 5D). 

The mitotic rate was high, and several atypical mitotic figures were observed. The tumour exhibited moderate differentiation, characterised by infiltrative nests of malignant epithelial cells displaying marked cellular polymorphism and nuclear atypia, yet maintaining partial squamous architectural features. These findings supported the final diagnosis of invasive squamous cell carcinoma (SCC) with features mimicking chronic osteomyelitis.

Based on these results, the patient was referred to the Oral Maxillofacial Department of King Fahad Medical City for further assessment and surgical management. Computed tomography (CT) of the head and neck revealed a right gingival lesion that demonstrated enhancement post-contrast, measuring 1.5×0.8×0.9 cm. The regional areas were distracted and involved the right mandibular body. The mandibular canal appeared to be separate from the lesion, and no other lesions were observed. The salivary glands were grossly within normal limits. The arterial phase revealed multiple atherosclerotic changes in the right common carotid artery, resulting in mild to moderate stenosis. There were also atherosclerotic changes in both carotid bifurcations without significant stenosis. CT of the head, neck, and facial bones showed an interval increase in the size of the right gingival and buccal space, enhancing the mass infiltrating the right buccinator muscle with osseous erosions of the buccal cortex of the right hemimandible. Osseous erosion appeared to abut the right mental canal, with questionable minimum interval dilation. The lesion appeared to encroach on the right retromolar trigone. The masses measured 3.4 cm in length and about 2.2 cm in depth.

**Figure 6:**
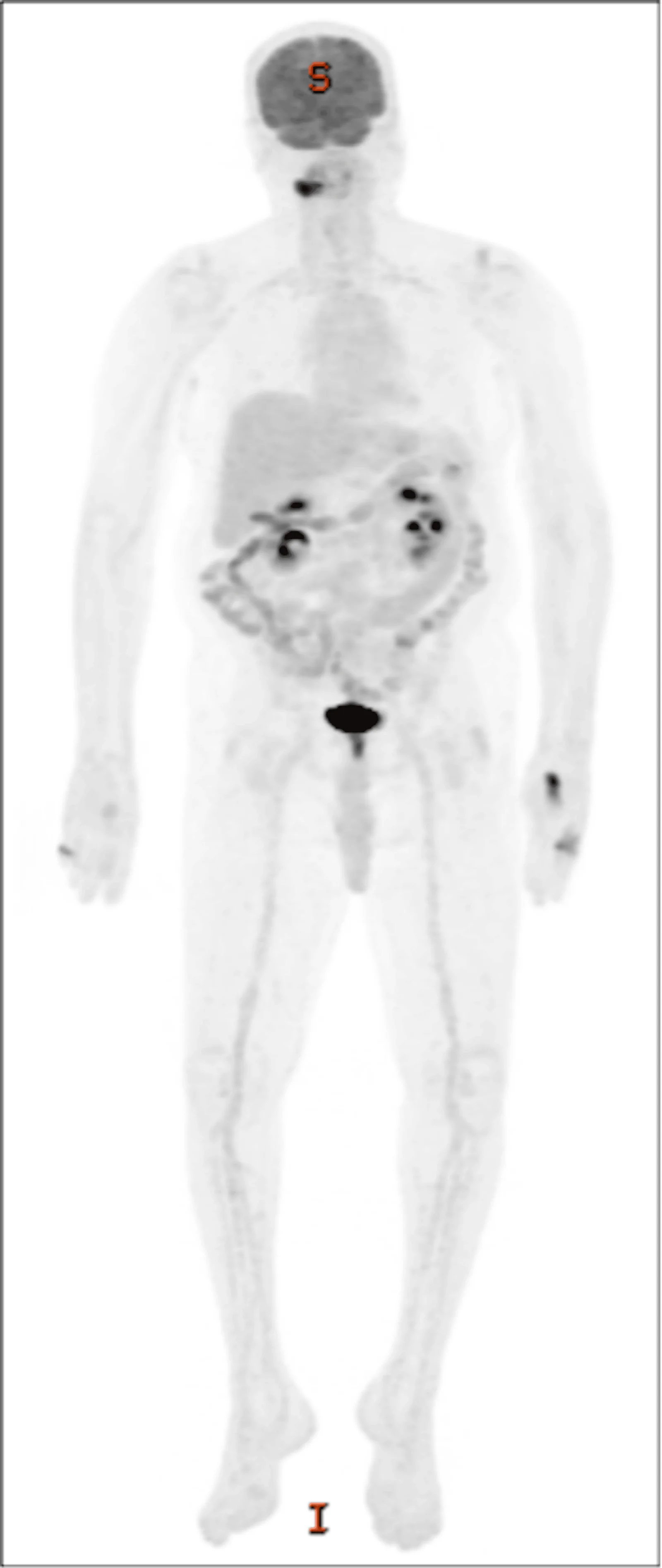
A whole-body positron emission tomography image depicting the avidity (hot spot) at the right mandible

**Figure 7:**
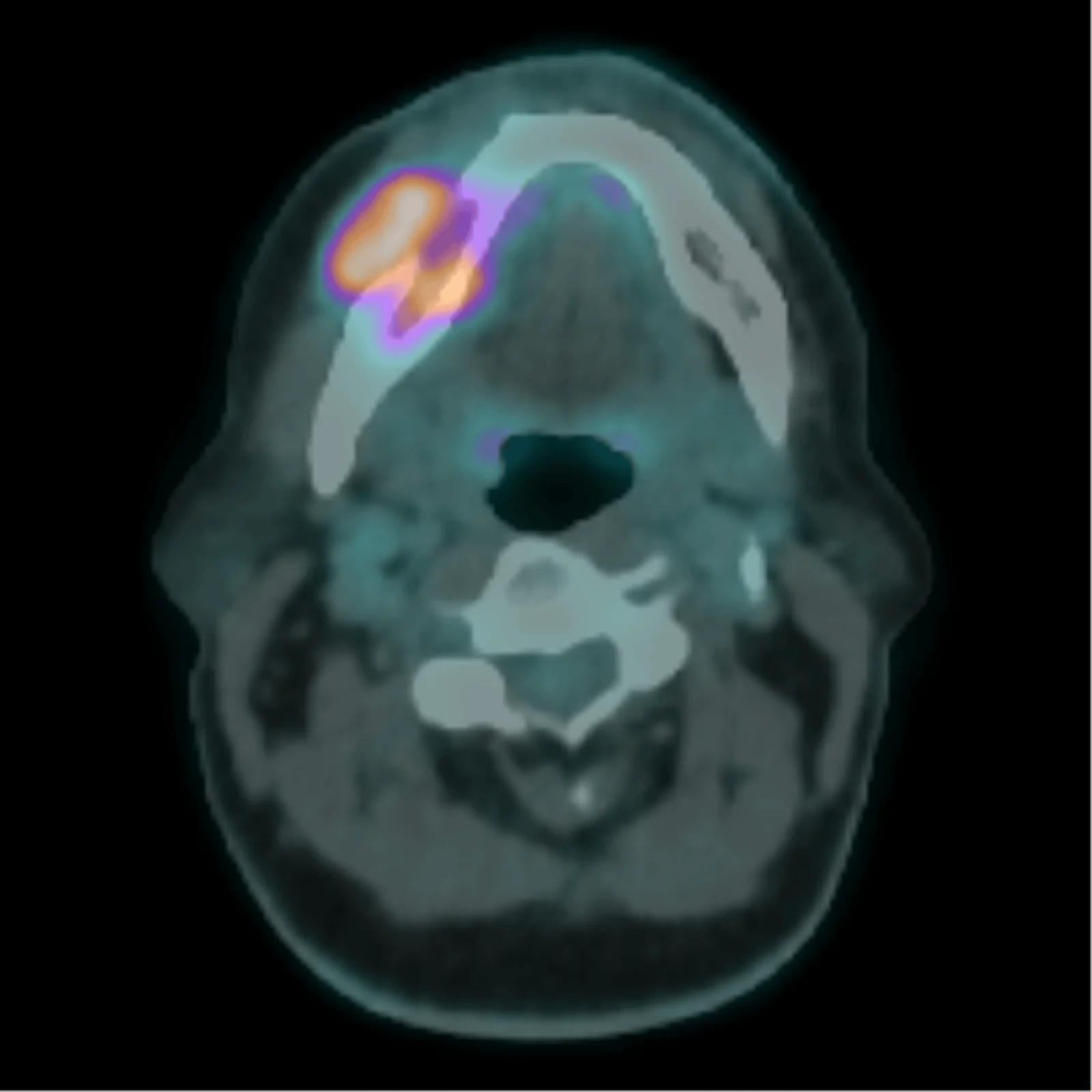
Positron emission tomography/computed tomography image (axial view) depicting the hot spot in the right mandibular molar region

Furthermore, suspicious enhancement of bilateral level 1 B lymph nodes was noted. The fluorine-18-fluorodeoxyglucose positron emission tomography (^18^F-FDG PET) of the whole body showed evidence of fluorodeoxyglucose (FDG) avidity in the known right mandibular SCC, associated with a bilateral, mildly FDG-avid cervical lymph node, but without FDG-avid distant metastasis (Figures 6-7).

The CT angiogram of the peripheral lower limbs and abdominal aorta showed that the abdominal aorta and common iliac artery were patent with calcified and noncalcified atherosclerotic changes, with no evidence of occlusion or aneurysmal dilatation.

Management and post-surgical assessment

**Figure 8:**
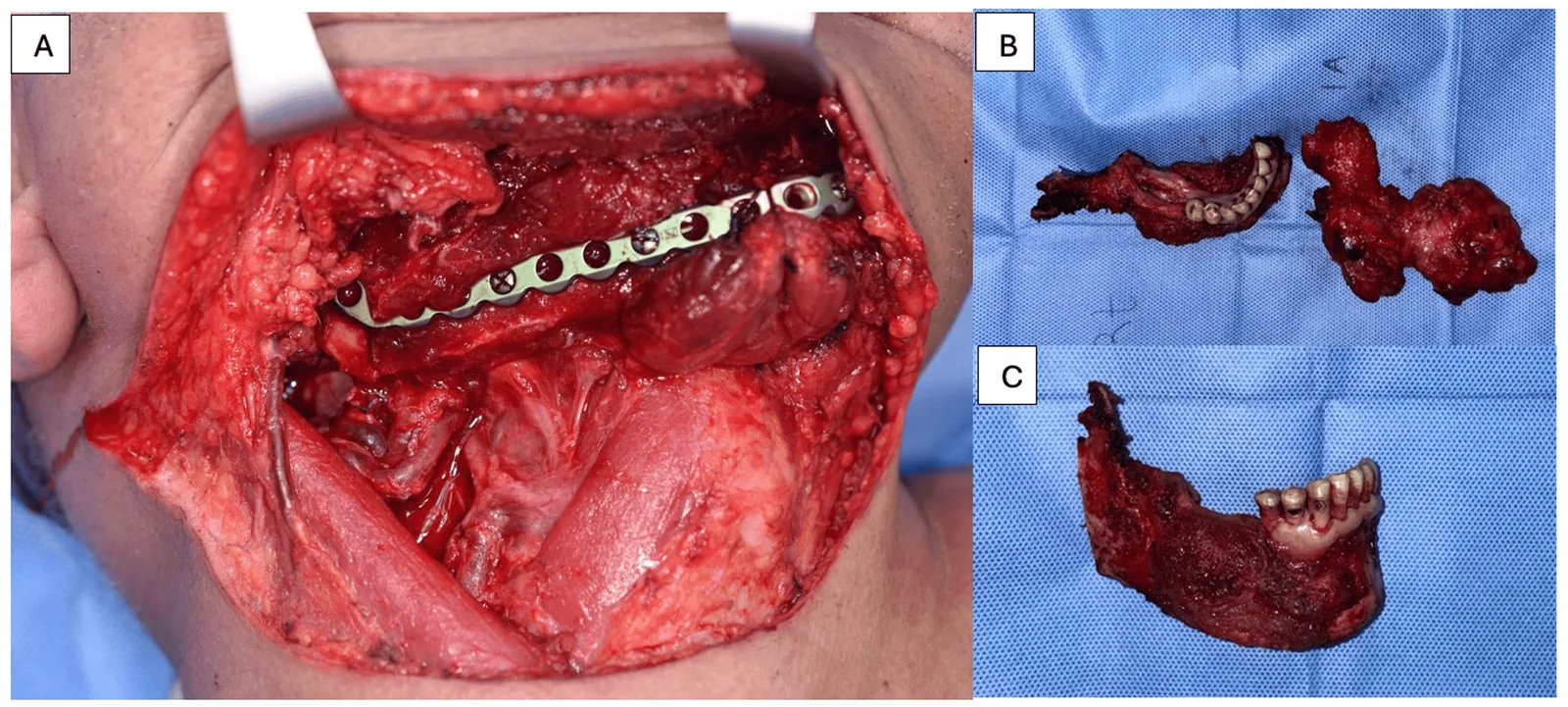
Resected specimens of (A) segmental mandibulectomy and ipsilateral neck dissection specimen levels 1-5 and (B-C) intraoperative view after tumour resection with reconstruction using a fibula free-flap secured to a reconstruction plate

The patient, staged as clinical cT4a cN0, underwent surgical treatment, including tracheostomy, mandibulectomy, right elective neck dissection, and reconstruction using a free fibula osteocutaneous flap (Figure 8A-8B).

This study was based on the recommendations of a multidisciplinary tumour board (MDTB) and in accordance with the National Comprehensive Cancer Network guidelines for advanced-stage oral cavity cancer.

The final pathological examination revealed a moderately differentiated SCC (grade G2), measuring 2.5 cm in diameter with a depth of invasion of 22 mm. Bone invasion was confirmed by categorising the tumour as stage pT4a. Although lymphovascular invasion was not identified, perineural invasion was observed. All 32 regional lymph nodes tested negative for cancer (pN0), indicating that there was no spread to the lymphatic tissue. However, invasive carcinoma was detected at the lateral deep soft tissue margin, and high-grade dysplasia was noted at the posterior mucosal margin. The patient subsequently underwent adjuvant chemoradiation per the MDTB recommendations and in accordance with the National Comprehensive Cancer Network Guidelines for Head and Neck Cancers, given the advanced stage of the disease and positive surgical margins [8]. The patient underwent surgical treatment including tracheostomy, mandibulectomy, right elective neck dissection, and reconstruction using a free fibula osteocutaneous flap with immediate dental implants placed into the fibular graft for planned future implant-supported dental rehabilitation.

**Figure 9:**
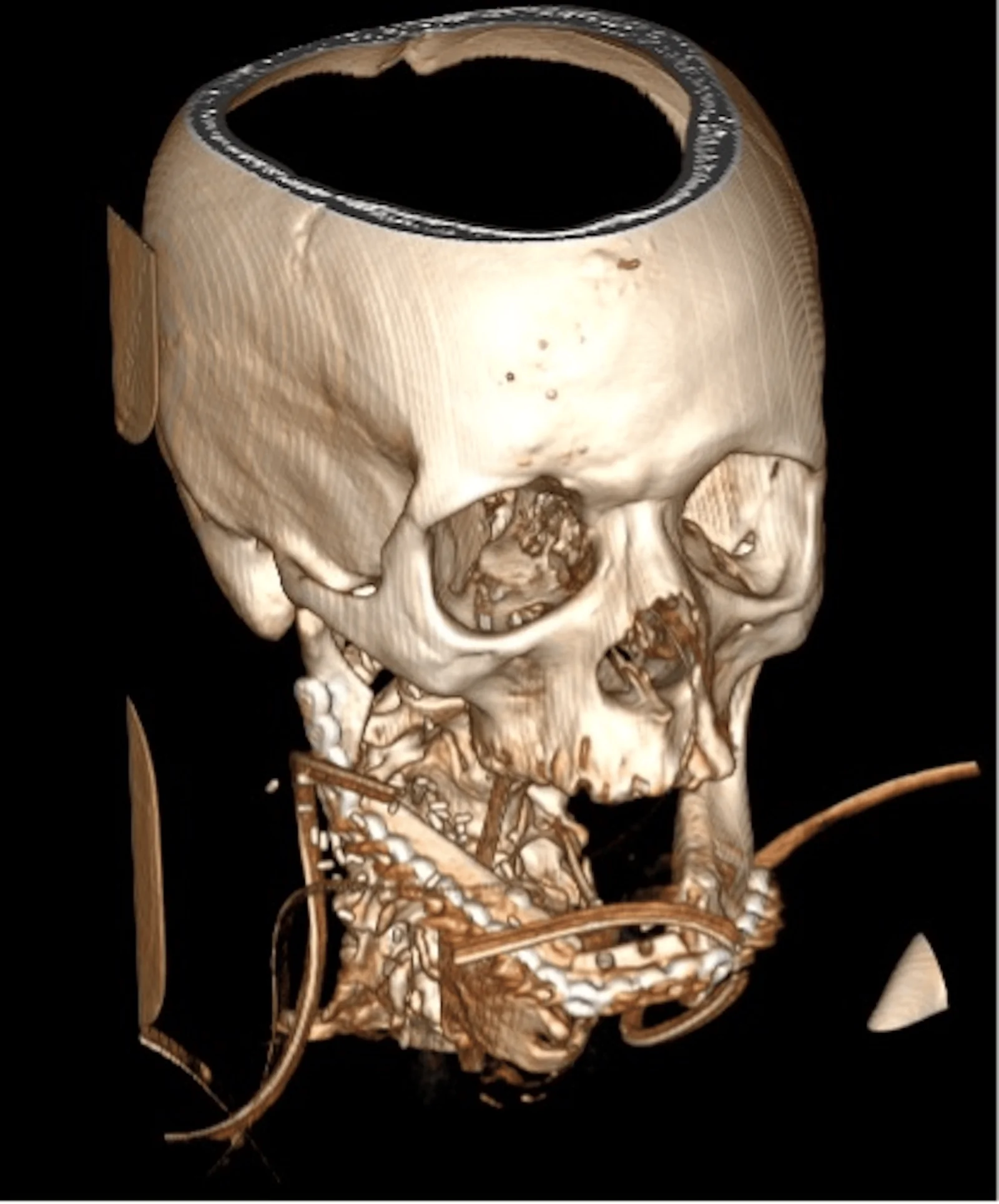
Post-surgical three-dimensional volume render depicting the extensive removal of the right and anterior mandible (hemimandibulectomy), sparing the condylar head. New bone grafts with fixation plates and screws are present

A CT of the head, neck, and facial bones showed postoperative changes in soft tissue oedema on the floor of the mouth and the right submandibular space along the right neck dissection bed. The fibular flap was aligned with the left hemimandible. The scan also revealed a mild inferior subluxation of the right temporomandibular joint. Remarkably, there is no evidence of residual lesions. Diagnosis and treatment initiation were delayed during the patient's initial presentation, highlighting the challenges within the diagnostic and treatment pathways (Figure 9).

Periodic follow-up assessments were planned to monitor progress, disease outcomes, and possible adverse complications of surgical management. He was also referred to maxillofacial prosthodontics for orofacial functional and aesthetic care plans.

## Discussion

The clinical features of OSCC and chronic osteomyelitis often overlap and can act as determinants for early recognition and diagnosis [4]. This diagnostic delay and late presentation could compromise short- and long-term prognoses, as observed in a previous case report in which the patient presented at an advanced stage with a prolonged history of jaw pain and radiographic evidence of an osteolytic lesion [9]. Similarly, Caruso et al. reported a case in which chronic osteomyelitis persisted for years before transforming into SCC [10]. In the literature review of case presentations, Ghosh et al. described a case with sinus tracts and draining pus [9], while Caruso et al. reported a persistent cutaneous fistula [10]. In contrast, our patient primarily presented with jaw pain after tooth extraction.

Histopathologically, our case and that of Ghosh et al. [9] were diagnosed as moderately differentiated SCC. In contrast, Jiang et al. reported well-differentiated SCC, highlighting the variation in tumour aggressiveness and prognosis [11]. However, definitive diagnosis should always be confirmed through biopsy, which in our case was invasive SCC [12]. Similarly, Caruso et al. described a persistent osteolytic lesion that was ultimately diagnosed as malignant [10]. The hallmark features of SCC observed in our case included keratin pearls, cellular pleomorphism, and hyperchromatism, along with infiltrative epithelial nests and evidence of stromal invasion, findings consistent with those reported for mandibular SCC [13]. Also, the persistence of inflammation has been demonstrated previously, and chronic osteomyelitis is believed to transform into SCC eventually [11]. The exact mechanism is not fully understood, but possible factors include chronic inflammation acting as a carcinogenic promoter, sustained epithelial turnover with accumulating genetic alterations (e.g., p53), and poorly vascularised scar tissue that increases mutagenic susceptibility while impairing immune surveillance [9,10]. This is distinct from our case, where OSCC is likely mimicking osteomyelitis at the initial presentation rather than arising from a pre-existing infection.

Imaging plays a crucial role in narrowing the differential diagnosis to determine the extent and complexity of a lesion, thereby guiding patient management. In line with the present study, Harmon et al. noted that the initial CBCT and MRI findings were initially recognised as osteomyelitis, as they often exhibited similar, sometimes indistinguishable, radiographic findings characterised by a moth-eaten appearance on plane and panoramic radiographs [12]. Previous imaging reports demonstrated extensive bicortical expansion, alveolar erosion, and soft tissue invasion beyond the primary osseous site, underscoring the aggressive nature of SCC [10]. By contrast, our imaging findings demonstrated localised bone invasion with cortical disruption.

The diagnostic delays of OSCC are often multifactorial, spanning approximately five months from symptom onset to definitive diagnosis. For instance, patient-related factors (e.g., low health literacy and difficulty in recognising the severity of symptoms) may contribute to the delayed seeking of medical attention. Healthcare system-related factors further contributed to this delay, including the initial misdiagnosis of osteomyelitis, lack of multidisciplinary collaboration, and prolonged referral pathways between private and specialised hospitals. Limited access to timely specialist evaluations and the absence of a coordinated liaison between disciplines have resulted in a protracted diagnostic journey, ultimately delaying the initiation of appropriate treatment [4].

Early detection of oral cancer is one of the most effective means for reducing its high mortality rate and minimising morbidity. Advanced stages often lead to severe functional impairment, facial disfigurement, psychological distress, and reduced quality of life. Therefore, early diagnosis and intervention are crucial [14]. Reducing diagnostic delays in OSCC requires improved clinical diagnostic skills and greater awareness of time as a critical factor in patient outcomes [15,16]. Enhancing OSCC education in medical and dental training and integrating telemedicine can aid in the early detection and monitoring of high-risk patients [17,18].

Clinicians can also follow the evidence-based guidelines (e.g., the American Dental Association's Living Clinical Practice Guideline) for the early detection of oral cancer and precancerous changes [17]. This includes obtaining a comprehensive medical, dental, and social history and conducting thorough extraoral and intraoral visual and tactile examinations. Any lesion or abnormality that persists without a definitive diagnosis or fails to resolve after 10-14 days despite appropriate management should be promptly biopsied for tissue diagnosis. In cases presenting as chronic osteomyelitis of the mandible, malignant transformation should be strongly suspected when a sinus tract or non-healing lesion persists for more than three years or when associated skin or soft tissue changes are observed. Implementing this evidence-based approach in patients with persistent mandibular pain, swelling, non-healing extraction sites, unexplained tooth mobility, paresthesia, or features indicative of chronic osteomyelitis may facilitate the earlier diagnosis of OSCC, reduce delays in treatment, and improve patient outcomes [17,18]. Additionally, improving doctor-patient communication is crucial for delivering prognostic discussions effectively [19]. Standardised diagnostic protocols can expedite referrals to specialised centres where multidisciplinary teams improve diagnosis, treatment, and patient outcomes.

## Conclusions

This case report highlights the significant diagnostic challenges associated with OSCC of the alveolar bone, particularly when its clinical and radiographic presentation mimics chronic osteomyelitis. For early recognition and intervention, imaging plays a vital role in initial assessments, along with histopathological assessments, when questionable signs and symptoms arise.
